# Functional evaluation of five *BRCA2* unclassified variants identified in a Sri Lankan cohort with inherited cancer syndromes using a mouse embryonic stem cell-based assay

**DOI:** 10.1186/s13058-020-01272-z

**Published:** 2020-05-11

**Authors:** Nirmala Sirisena, Kajal Biswas, Teresa Sullivan, Stacey Stauffer, Linda Cleveland, Eileen Southon, Vajira H. W. Dissanayake, Shyam K. Sharan

**Affiliations:** 1 Human Genetics Unit, Faculty of Medicine, University of Colombo, Colombo, 8 Sri Lanka; 2 Mouse Cancer Genetics Program, Center for Cancer Research, National Cancer Institute, Bldg 560, Room 32-33, 1050 Boyles Street, Frederick, MD 21702 USA

**Keywords:** *BRCA2*, Classification, Functional assay, Inherited cancer, Next-generation sequencing, Variants of unknown clinical significance (VUS)

## Abstract

Next-generation sequencing of Sri Lankan families with inherited cancer syndromes resulted in the identification of five *BRCA2* variants of unknown clinical significance. Interpreting such variants poses significant challenges for both clinicians and patients. Using a mouse embryonic stem cell-based functional assay, we found I785V, N830D, and K2077N to be functionally indistinguishable from wild-type *BRCA2*. Specific but mild sensitivity to olaparib and reduction in homologous recombination (HR) efficiency suggest partial loss of function of the A262T variant. This variant is located in the N-terminal DNA binding domain of BRCA2 that can facilitate HR by binding to dsDNA/ssDNA junctions. P3039P is clearly pathogenic because of premature protein truncation caused by exon 23 skipping. These findings highlight the value of mouse embryonic stem cell-based assays for determining the functional significance of variants of unknown clinical significance and provide valuable information regarding risk estimation and genetic counseling of families carrying these *BRCA2* variants.

Breast cancer is the most common cancer in women and a leading cause of cancer morbidity and mortality in Sri Lanka [1]. Latest epidemiological reports indicate that breast cancer accounts for 13.1% of all cancers and 24% of all female cancers in the country [2]. These figures highlight the importance of identifying individuals at risk of breast cancer early so that appropriate management and preventive measures could be undertaken to reduce the morbidity and mortality associated with this disease. The advent of next-generation sequencing (NGS)-based genomic testing has facilitated rapid, precise genetic diagnosis and management of patients with inherited cancer syndromes.

In 2015, using the Illumina MiSeq NGS platform and an in-house developed validated bioinformatics pipeline, multi-gene cancer panel testing and clinical exome sequencing were successfully implemented at our center for the genetic evaluation of patients with inherited cancer syndromes [3, 4]. However, the implementation of NGS-based genomic testing into our routine clinical cancer practice has simultaneously yielded a multitude of rare germline variants in cancer predisposing genes that are known as variants of unknown clinical significance (VUS). This poses significant challenges for both patients and clinicians, especially with regard to risk assessment, genetic counseling, and clinical decision making. Such variants might not contribute to risk assessment and may at times prompt anxiety and overtreatment. In this regard, the non-representation of genetic variants found in the Sri Lankan population in public databases is an additional drawback and challenge. To overcome this limitation, often times we resort to careful assessment of the three-generation pedigrees and testing the particular variant in other affected and unaffected family members, for further confirmation and to identify a clear pattern of co-segregation in the family members. However, the only means to precisely delineate the exact biological significance of these variants is through functional studies. This study aims to describe the functional assays which were conducted to determine the functional significance of five VUS identified in Sri Lankan families with inherited cancer syndromes.

We retrospectively analyzed the clinical and genetic test data of consecutive patients from families with two or more patients with inherited cancer syndromes who underwent NGS-based testing between January 2015 and December 2018 which were maintained prospectively in a database. Ethical clearance for the study was obtained from the Ethics Review Committee of the Faculty of Medicine, University of Colombo [EC-13-182]. Written informed consent was obtained from all the study participants. The genetic variants were classified using the five-class system as pathogenic, likely pathogenic, VUS, likely benign, or benign according to the lab classification criteria. This criteria relies on the guidelines of the American College of Medical Genetics and Genomics (ACMG) and the Association of Molecular Pathology [5]. All retained variants underwent thorough assessment and review of available evidence (e.g., population frequency databases, published literature, case/control and functional studies, internal co-occurrence and co-segregation data, evolutionary conservation, and in silico functional predictions) to arrive at a final variant classification. The variants in the *BRCA1* and *BRCA2* genes identified in this cohort are summarized in Table 1. We focused our studies on five VUS identified in the *BRCA2* gene in this cohort for further investigations to determine their functional significance using a mouse embryonic stem (mES) cell-based assay.

**Table 1:** Summary of *BRCA1* and *BRCA2* variants identified in Sri Lankan families with inherited cancer syndromes

**Variant**	**Amino acid change**	**ClinVar interpretation**	**Cancer types in index cases**	**Cancer types in family members**
*BRCA1:*c.1575del	p.Gln526Lysfs	Pathogenic	Breast	Breast, ovarian, endometrial
*BRCA1:*c.3392A>G	p.Asp1131Gly	VUS	Breast	Breast
*BRCA1:*c.4120_4121delAG	p.Ser1374Terfs	Pathogenic	Breast	Breast, thyroid
*BRCA1:*c.5289delG	p.Leu1764Terfs	Pathogenic	Breast, ovary	Breast, endometrial, ovarian, thyroid, hepatic, esophageal
*BRCA1:*c.68_69delAG	p.Glu23Valfs	Pathogenic	Ovary	Breast
*BRCA1:*c.1881_1884del	p.Ser628fs	Pathogenic	Breast	Breast, colorectal
*BRCA2:*c.784G>A	p.Ala262Thr	VUS	Breast and ovarian	Breast, thyroid, and endometrial
*BRCA2:*c.2353A>G	p.Ile785Val	VUS	Prostate	Colorectal and thyroid
*BRCA2:*c.2488A>G	p.Asn830Asp	VUS	Breast	Ovarian
*BRCA2:*c. 6231G>C	p.Lys2077Asn	Likely Benign/VUS	Breast	Breast
*BRCA2:*c.9117G>A	p.Pro3039=	Pathogenic	Breast	Breast, thyroid, and endometrial
*BRCA2:*c.5727_5728insG	p.Asn1910fs	Pathogenic	Ovary	Ovarian, liver, colon, prostate
*BRCA2:*c.1296_1297delGA	p.Asn433Glnfs	Pathogenic	Breast, fallopian tube	Breast, liver, endometrial, colorectal, ovarian, esophageal
*BRCA2:*c.5576_5579delTTAA	p.Ile1859Lysfs	Pathogenic	Breast	Breast, endometrial, gastric
*BRCA2:*c.5621_5624delTTAA	p.Ile1874Argfs	Pathogenic	Breast	Breast, ovarian

Mouse embryonic stem (mES) cell-based assays provide a simple and reliable assay to test the functional significance of *BRCA2* VUS [6]. The assay is based on the observation that BRCA2 is essential for mES cell viability. The ability of human BRCA2 to rescue the lethality of *Brca2*-deficient mES cells and the sensitivity of viable cells to various DNA damaging agents are used to evaluate the functional significance of the variants [6]. We used this approach to determine the functional significance of five unclassified *BRCA2* germline variants [NM_000059.3:c.784G>A|NP_000050.2:p.Ala262Thr|rs397507393; NM_000059.3:c.2353A>G|NP_000050.2:p.Ile785Val|rs747748537; NM_000059.3:c.2488A>G|NP_000050.2:p.Asn830Asp|rs574039421; NM_000059.3:c.6231G>C|NP_000050.2:p.Lys2077Asn|rs541826447;

NM_000059.3:c.9117G>A|NP_000050.2:p.Pro3039Pro|rs28897756] identified in Sri Lankan families with hereditary breast and ovarian cancer syndrome (Fig. 1a).

**Fig. 1:**
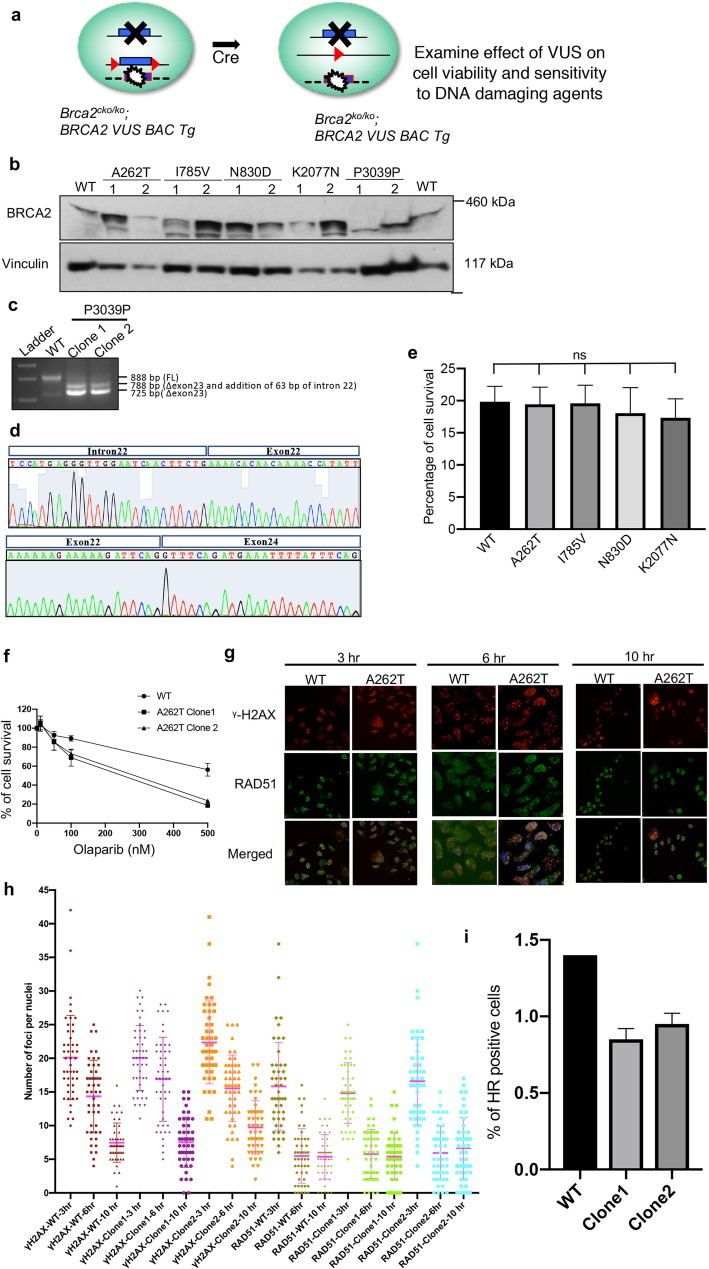
Functional analysis of *BRCA2* variants in *Brca2*^*cko/ko*^ mES cells*.***a** Schematic representation of the mES cell-based functional assay. **b** Expression of *BRCA2* variants in mES cells by Western blotting. Two independent clones were generated for each variant. Vinculin was used as loading control. **c** Expression of BRCA2 P3039P by RT-PCR using primers from exons 20 (5′-AGGAAGAAAAGGAAGCAGCAAAATATGTGG-3′) and 25 (5′-TCTCCAGCAAATAAAGTAAGAAGG-3′) revealed lack of full-length transcript. Two alternatively spliced transcripts were observed. **d** Sequence analysis of the two transcripts revealed major alternatively spliced transcript skipped exon 23 (lower band) and a minor form that skipped exon 23 but retained 63pb of intron 22 (upper band). **e** Quantification of viability of *Brca2*^*ko/ko*^ mES cell by various *BRCA2* variants. Two independent BAC clones expressing the variants were analyzed. Average numbers of viable cells from two independent clones were plotted. mES cell clone expressing WT BRCA2 was used as control. **f** Clonogenic survival assay confirming sensitivity of mES cells expressing A262T to olaparib (*P* value < 0.001 at 100 nM and < 0.0001 at 500 nM concentrations using a multiple *t*-test). **g** Representative images showing γH2AX and RAD51 foci at 3, 6, and 10 h post 10 Gy IR in mES cells expressing WT and A262T BRCA2. **h** Quantification of γH2AX and RAD51 foci at 3, 6, and 10 h post 10 Gy IR in mES cells expressing WT and A262T BRCA2. **i** Quantification of homologous recombination using a GFP-based HR reporter in mES cells expressing WT and A262T BRCA2 (*P* values are 0.008 for Clone 1 and 0.01 for Clone 2 using paired *t*-test)

The desired *BRCA2* variants were generated in bacterial artificial chromosomes (BAC) and expressed in mES cells (Fig. 1b). We failed to detect the full-length protein expression of BRCA2 p.Pro3039Pro variant by Western analysis. This silent mutation at the coding level resulted in skipping of exon 23 and production of a smaller non-functional protein (Fig. 1c, d). This variant is known to disrupt a splice donor site and has previously been shown in in vitro studies to cause aberrant mRNA processing with skipping of exon 23 [7]. Our finding further corroborates the earlier observations and clearly establishes the pathogenicity of this variant. p.Pro3039Pro has previously been reported in families with breast and/or ovarian cancer [8–12].

The p.Ile785Val, p.Asn830Asp, and p.Lys2077Asn variants are likely to be neutral as they rescued mES cell lethality and were indistinguishable from wild-type (WT) BRCA2 in their sensitivity to DNA damaging agents such as cisplatin, camptothecin, mitomycin C, methyl methanesulfonate (MMS), olaparib (poly ADP-ribose polymerase inhibitor), and γ-irradiation (IR) as measured by XTT cell-proliferation assay (Fig. 1e, Table 2). Interestingly p.Ala262Thr was also indistinguishable from WT in all assays except it exhibited specific but mild sensitivity to olaparib among the drugs tested. This was further confirmed by clonogenic survival assay (Fig. 1f). We did not observe any defect in RAD51 recruitment at 3, 6, and 10 h after 10 Gy IR (Fig. 1g, h). However, using a green fluorescent protein (GFP)-based reporter assay [13], we observed a reduction in homologous recombination (HR) efficiency of p.Ala262Thr compared to WT BRCA2 (Fig. 1i) suggesting that RAD51 foci formation may not be sensitive enough to detect minor reduction in HR efficiency.

**Table 2:** Sensitivity of mES cells expressing *BRCA2* variants to different DNA-damaging agents

**Variants**	**MMS**	**Mitomycin C**	**Cisplatin**	**Camptothecin**	**Olaparib**	**IR**
A262T	No	No	No	No	Yes (mild)	No
I785V	No	No	No	No	No	No
N830D	No	No	No	No	No	No
K2077N	No	No	No	No	No	No
P3039P	NA	NA	NA	NA	NA	NA

*NA* not available, *mES* mouse embryonic stem cells, *MMS* methyl methanesulfonate, *IR* γ-irradiation

These findings highlight the value of mES cell-based assays for determining the functional significance of unclassified variants and further extends the spectrum of germline pathogenic variants in the *BRCA2* gene with functional evidence. Our findings revealed a mild and specific effect of p.Ala262Thr variant on sensitivity to olaparib as well as impact on HR. To date, most pathogenic mutations in BRCA2 have been identified in the C-terminal DNA binding domain of BRCA2 [14, 15]. A second DNA binding domain has been identified near the N-terminus of BRCA2 between residues 250 and 500, which contains a putative zinc-finger (zf) PARP-like domain between residues 265 and 349 [16]. DNA binding studies have shown its ability to bind to various DNA structures including ssDNA. Interestingly, unlike the C-terminal domain, it also exhibits dsDNA binding activity, which is predicted to facilitate the interaction of BRCA2 to dsDNA/ssDNA junctions during HR. Our preliminary studies with p.Ala262Thr variant did not reveal a defect in binding of BRCA2 to the chromatin (data not shown). Future studies will be aimed at understanding if p.Ala262Thr has any effect on RPA-dependent strand exchange ability of RAD51. A defect in RPA-dependent strand exchange will explain the specific effect of this variant on HR and olaparib sensitivity [16].

## Data Availability

The datasets used and/or analyzed during the current study are available from the corresponding author on reasonable request.

## Abbreviations

BAC: Bacterial artificial chromosomes
GFP: Green fluorescent protein
HR: Homologous recombination
IR: γ-irradiation
mES: Mouse embryonic stem
MMS: Methyl methanesulfonate
NGS: Next-generation sequencing
VUS: Variants of unknown clinical significance
WT: Wild-type
